# The Treatment of Sepsis

**Published:** 1910-03-19

**Authors:** 


### The Hospital

A Journal of

The Medical Sciences and Hospital Administration.

New Series. No. 161, Vol. VI. [No. 1233, Vol. XLVII.] SATURDAY, MARCH 19, 1910.

### The Treatment of Sepsis.

On a subsequent page of this issue appears the report of a post-graduate lecture on the Treatment of Septic Diseases, delivered by Mr. E. M. Corner. In this lecture are summarised the experiences of the author while in charge of the wards set apart at St. Thomas' Hospital for the reception of these cases, and his conclusions are very well worth the careful attention of every general practitioner, particularly in respect of those forms of treatment which have been recently introduced; these are still unfamiliar even to many whose medical education *in statu pupillari* has extended well into the twentieth century. Mr. Corner, indeed, begins by mentioning a concomitant, or rather an essential ingredient, of treatment whose value has been dinned steadily into every student since the days of Hilton, namely Rest. To the profession at large there is little need to eulogise this important factor in the treatment of sepsis, though the public is still often curiously reluctant to take advice in this sense and entirely sceptical of the necessity for it. Hot dressings, again, are no novelties; yet there is much regrettable indifference as to their application in such a way as to secure their very great therapeutic influence. In this connection it is often overlooked that a thick pad of very hot cyanide gauze preserves its character of a hot dressing much better than the time-honoured boracic lint fomentation; it is, unfortunately, much more expensive, and in this connection it is well to note that any hot fomentation should be changed at intervals not any longer than four hours.

There are, however, two new methods of treatment which have been widely extolled for septic infections; they are known as Bier's hyperæmic treatment and vaccine therapy. Both have been highly, even extravagantly, praised by those who have been amongst the first or the most active in adopting them: both have failed in some measure to justify the enthusiasm of their most thorough-going supporters. They have now been under trial sufficiently long for a more judicial estimation of their merits than is usually possible in the early days of any much-advertised remedy. Such an estimate Mr. Corner supplies; and it will probably surprise many to find that of the two he has had more success in the congestion treatment than with the use of vaccines. In the elastic bandage and the suction glass it would seem that a principle has been introduced into surgery which, used with discretion, must be considered a distinct and encouraging advance. Yet it must also be recognised that the limitations of Bier's method are numerous to suppose that the incision of abscesses and such like established procedures are superseded is quite erroneous and can only result in the discredit of the hyperæmic treatment. It will be noted that, while Mr. Corner utters much needed words of warning in this sense, he is also strongly of opinion that incisions may with safety and advantage be made much smaller when they are to be followed by passive congestion. He also rightly emphasises the care which must be paid to correct adjustment of the bandage: the patient's sensations are the best guide to this, and the co-operation of an intelligent patient to whom the importance of his frank opinion has been explained is of very great help. In certain cases failure has been the result: of these, as is pointed out, erysipelas is pre-eminent, and for this troublesome disease passive congestion is better avoided.

The literature of the vaccine treatment of bacterial infections in general, and of septic diseases in particular, contains many accounts of remarkable recoveries and but few of disappointing failures. That this is due partly at least to the non-publication of the second class may be assumed, but whether success or disappointment is the more common the profession at large has very little chance of correctly judging. It is of interest to read that at St. Thomas's Hospital vaccines have proved of no very great assistance in dealing with septic cases, and that the results now obtained are much the same as before the introduction of this form of treatment. It is also curious that cases have been known in which the administration of "stock" vaccines have been followed by improvement not obtained when cultures of the patients' own organisms have been employed. On the whole, it may be justly said that vaccine therapy in septic infections has not invalidated any one of the surgical canons that have been established during the last quarter of a century. It is to be remembered that antistreptococcic sera, which have fallen somewhat out of fashion since the high hopes engendered by vaccine therapy were formed, are probably just as valuable as the newer vaccines; and it is also important to bear in mind that quite a fair percentage of apparently hopeless septicæmic and pyæmic cases recovered when both these bacteriological products were undreamed of. Mr. Corner's paragraphs on the relation between sepsis and glycosuria draw attention to an association which might well be made the subject of a careful research. Everyone knows the tendency to carbuncle which diabetics not uncommonly exhibit; but the converse, glycosuria following on a septic disease, is not nearly so much commented on by teachers and textbooks. Nevertheless, there is no doubt that this curious sequence does occur; possibly it goes fairly often undetected. It is at least a matter to be kept in mind when dealing with a patient much debilitated by a long-standing and exhausting form of sepsis; and it is important to distinguish between this form of glycosuria and true antecedent diabetes, for the distinction may materially affect the treatment.

